# Cage positioning as a risk factor for posterior cage migration following transforaminal lumbar interbody fusion – an analysis of 953 cases

**DOI:** 10.1186/s12891-019-2630-0

**Published:** 2019-05-29

**Authors:** Yung-Hsueh Hu, Chi-Chien Niu, Ming-Kai Hsieh, Tsung-Ting Tsai, Wen-Jer Chen, Po-Liang Lai

**Affiliations:** 1 Department of Orthopedic Surgery, Chang Gung Memorial Hospital, No. 5, Fuxing St., Guishan Dist., Taoyuan City, Linkou 33305 Taiwan; 2 Bone and Joint Research Center, Chang Gung Memorial Hospital, No. 5, Fuxing St., Guishan Dist., Taoyuan City, Linkou 33305 Taiwan; 3 College of Medicine, Chang Gung University, No.259, Wenhua 1st Rd., Guishan Dist, Taoyuan City, 33302 Taiwan; 4 Department of Orthopedic Surgery, Chung Shan Hospital, No.11, Ln. 112, Sec. 4, Ren’ai Rd., Da’an Dist, Taipei City, 10689 Taiwan

**Keywords:** Transforaminal lumbar interbody fusion (TLIF), Posterior cage migration, Cage positioning, Risk factor, Multivariate analysis

## Abstract

**Background:**

The risk of posterior cage migration (PCM) exists when a fusion cage is used for transforaminal lumbar interbody fusion (TLIF). This complication is influenced by contact pressure between the endplate and the cage. Previous reports demonstrated that anteriorly located cages bore more load and had greater strain than posteriorly located cages. However, there have been no detailed reports on the correlation between cage positioning and PCM.

**Methods:**

From March 2014 to October 2015, we reviewed 953 patients receiving open transforaminal lumbar interbody fusion (TLIF) and bilateral pedicle screw instrumentation. One hundred patients without PCM were randomly sampled as the control group. Postoperative sagittal and coronal cage positions in the disc space were evaluated with the ‘depth ratio’ and the ‘coronal ratio’. The demographic data of patients with and without PCM were compared to detect patient-related factors. Radiographic and cage related parameters, including cage position, preoperative disc height, preoperative spine stability, cage geometry, cage size, and height variance (= cage height – preoperative disc height) were compared between the PCM group and the control group. Univariate analyses and a multivariate logistic model were used to identify risk factors of PCM.

**Results:**

Posterior cage migration occurred in 24 (2.52%) of 953 patients. The univariate and multivariate analyses revealed that those with a decreased depth ratio (OR, 9.78E-4; 95% CI, 9.69E-4 – 9.87E-4; *p* < 0.001) and height variance (OR, 0.757, 95% CI, 0.575–0997, *p* = 0.048) had a significantly higher risk of developing PCM.

**Conclusions:**

Our results verified that posteriorly located cages and undersized cages are more prone to developing PCM, which may aid surgeons in making optimal decisions during TLIF procedures.

## Introduction

Transforaminal interbody fusion was Initially described by Harms and Rolinger in the early 1980’s, it has become a widely accepted procedure for patients with degenerative spine diseases [1]. The fusion cage theoretically provides spinal stability, anterior column support, and restoration of the disc space height and the neuroforaminal area. However, the possibility of postoperative posterior cage migration (PCM) into the spinal canal or neuroforamen exists. This migration might result in direct compression of nerve roots, instrumentation failure, or revision operation [2–4].

To prevent PCM, the contact pressure between the cage and the endplate should generate enough friction to resist the posterior migration force. A biomechanical study performed by Polly et al. demonstrated that cage strain increased significantly as cages were placed more anteriorly. Compared to constructs with posterior cage placement, constructs with anterior cage placement were found to have greater stiffness on axial compression [5]. It was theorized that cages placed in anterior disc space shared more load and provided more anterior column support. Previous reports from Aoki et al. reported that cages located more posteriorly to posterior margin of either cranial or caudal endplates had non-significant higher rate (*p = 0.068*) of PCM. Based on these findings, we hypothesized that cage positioning in disc space is an important factor in PCM.

To our knowledge, few clinical studies have researched the relation of cage positioning and PCM. Prior published reports indicated several risk factors for PCM following posterolateral interbody fusion (PLIF) and TLIF, including undersized cages, unilateral pedicle screw fixation, end disc fusion, bullet-type cages, pear-shaped discs, surgeon experience, and high preoperative disc height [3, 4, 6–10]. The impact of cage position on PCM has yet to be identified.

To verify our hypothesis, we conducted a retrospective study to address the relationship between cage position and PCM, and we also aimed to identify other risk factors for PCM.

## Materials and methods

### Patients

We retrospectively reviewed patients who were treated with open TLIF and combined bilateral pedicle screw instrumentation between March 2014 and October 2015. The study was approved by the Institutional Review Board of the Chang Gung Medical Foundation before the data collection and analyses (IRB 201800785B0). Medical records, clinical features, and preoperative and postoperative radiographs were reviewed. All the patients included in this study experienced pain or palsy derived from degenerative lumbar spinal diseases that were not relieved by conservative treatment. The indications for surgeries were categorized into 1) spondylolytic spondylolisthesis, 2) degenerative kyphosis or scoliosis, 3) degenerative spondylolisthesis, and 4) revision spine surgery. We excluded patients with postoperative follow-up of less than 24 months, or those with deep spine surgical site infection (SSI) requiring debridement surgery. Patients who underwent dynamic pedicle screw instrumentation or minimally invasive TLIF procedures were also excluded from this study.

### Surgical technique and equipment

A midline posterior approach was performed to expose the lamina and facet joints on both sides. Pedicle screws were then placed on both sides of the intended fusion levels. Laminectomy and bilateral medial facetectomy were done to achieve adequate decompression. Lateral recess or nerve root canal decompression was also performed when necessary. The cages were chosen to insert on the side with major neurological symptoms. After meticulous discectomy and thorough disc preparation, the autologous bone graft from resected bone was milled and packed into the disc space. The cage packed with the autologous bone graft was then inserted into the disc space. Additional bone substitutes or allograft were used in some cases if autologous bone graft was not sufficient. Postoperative radiographs were taken before wound closure to check implant position.

The cages used in this series were categorized into kidney-shaped designs, including the TSpace cage (Aesculap Inc., Center Valley, PA); and bullet-shaped designs, including the Capstone cage (Medtronic Sofamor Danek, Minneapolis, MN), OPAL cage (Synthes, West Chester, PA), Reborn essence cage (BAUI, Taipei, Taiwan), and Vigor cage (A-spine, Taipei, Taiwan). All the cages were radiolucent polyetheretherketone (PEEK) devices with radiopaque markers to identify the cage position.

### Radiographic assessment

We defined PCM as meeting both the following conditions: 1) radiopaque markers migrated posteriorly > 2 mm by comparing postoperative radiographs to the immediate postoperative radiograph; 2) the radiopaque marker touched or were located more posteriorly than the posterior margin of either vertebral endplate.

Standard standing anteroposterior (AP) radiographs, lateral radiographs, dynamic radiographs, and magnetic resonance imaging (MRI) were obtained before surgery. On the lateral radiographs, the percent of slippage, the range of motion (ROM) of the disc, and the sagittal translation were measured according to established methods [11, 12]. The preoperative disc height (DH) was measured on MRI as distance between midpoints of cranial and caudal endplates on a mid-sagittal cut [13]. We also obtained a value by subtracting preoperative DH from cage height and defined it as ‘height variance’ to represent the relative cage size compared to the disc space. Height variance was deemed positive if the cage height was larger than the measured preoperative disc height, and was deemed negative if the cage height was smaller than the preoperative disc height.

**Fig. 1:**
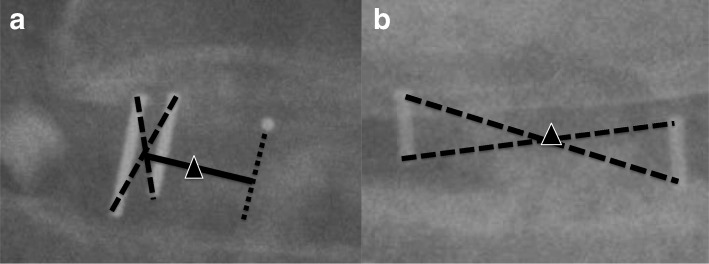
Methods of defining the cage center. **a** The triangle indicates the cage center of the kidney-shaped cage. **b** The triangle indicates the cage center of the bullet-shaped cage

**Fig. 2:**
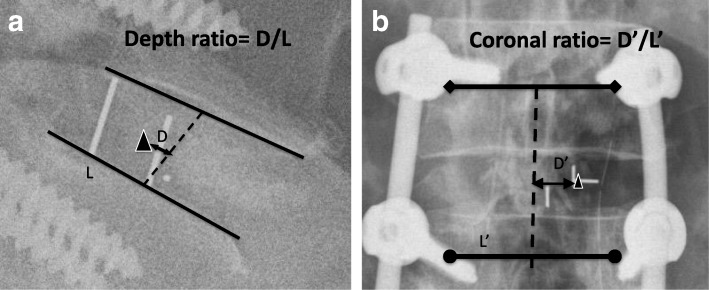
Measurement of the depth ratio and the coronal ratio. The triangle indicates the cage center. **a** Depth ratio = D/L. L (solid line) indicates caudal endplate length. D (double-arrowed line), parallel to L, is the distance between the cage center and the disc center. **b** Coronal ratio = D’/L’. L’ (solid line) indicates the distance between caudal pedicles. D’ (double-arrowed line), parallel to L’, is the distance between the triangle and the midline

To check the position of the cage, postoperative radiographs were obtained within 1 week, 1 month, 3 months, 6 months, and 12 months after surgery. Additional follow-up radiographs were obtained for patients with acute symptoms. Sagittal and coronal cage positions were recorded on immediate postoperative radiographs by measurement of the ‘depth ratio’ and the ‘coronal ratio’ respectively. The cage center on the radiograph was first defined. For the kidney-shaped cage with three radiopaque markers, a line parallel to radiopaque lines was first made from the radiopaque dot (Fig. 1a). The cage center was located on the midpoint between the line from the radiopaque dot and the geometric center of the two radiopaque lines. The cage center of the bullet-shaped cage with two radiopaque markers was located on the geometric center of two radiopaque lines (Fig. 1b). The depth ratio was measured on lateral radiograph as the distance between the cage center and the disc center (connection between midpoints of cranial and caudal endplates) divided by the caudal endplate length (Fig. 2a). The value was deemed positive for cages located more anteriorly than the disc center and negative for cages located more posteriorly than the disc center. The coronal ratio was measured by calculating the distance of the cage center from the midline (connection between midpoints of the cranial segment pedicles and the caudal segment pedicles) divided by the distance between pedicles of the caudal segment on postoperative AP view (Fig. 2b). The value was deemed positive if the cage was located across the midline from insertion side and negative for cages located at the insertion side.

To compare the radiological findings between patients with and without PCM, 100 patients were randomly selected as the control group from 929 patients without PCM. The random samples were collected via the random sampling add-ons in Microsoft Excel 2016.

### Data analysis

We first compared demographic data between patients with and without PCM to identify patient factors related to PCM, followed by comparing radiographic parameters between migrated cages in the PCM group and non-migrated cages in the control group to detect cage- or disc-related factors of PCM. We also compared migrated cages and non-migrated cages in the PCM group to exclude the influence of patient factors.

Independent t tests was performed to analyze differences in continuous variables. A Pearson’s Chi-square test or Fisher’s exact test was used to examine the differences among categorical variables. Variables with *p*-values < 0.05 in the univariate analyses were entered into a multivariate logistic regression model. SPSS statistical software version 20 (IBM Corporation, Armonk, NY, USA) was used to perform the statistical analyses. Differences were deemed significant if the *p*-value was < 0.05.

## Results

### Prevalence, and characteristics of PCM among 953 patients

**Fig. 3:**
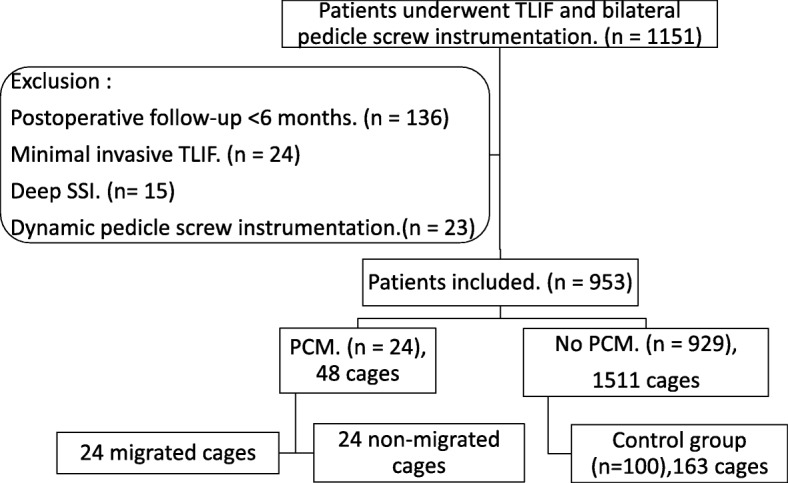
Flow diagram of study groups. TLIF: transforaminal lumbar interbody fusion. SSI: surgical site infection. PCM: posterior cage migration

**Fig. 4:**
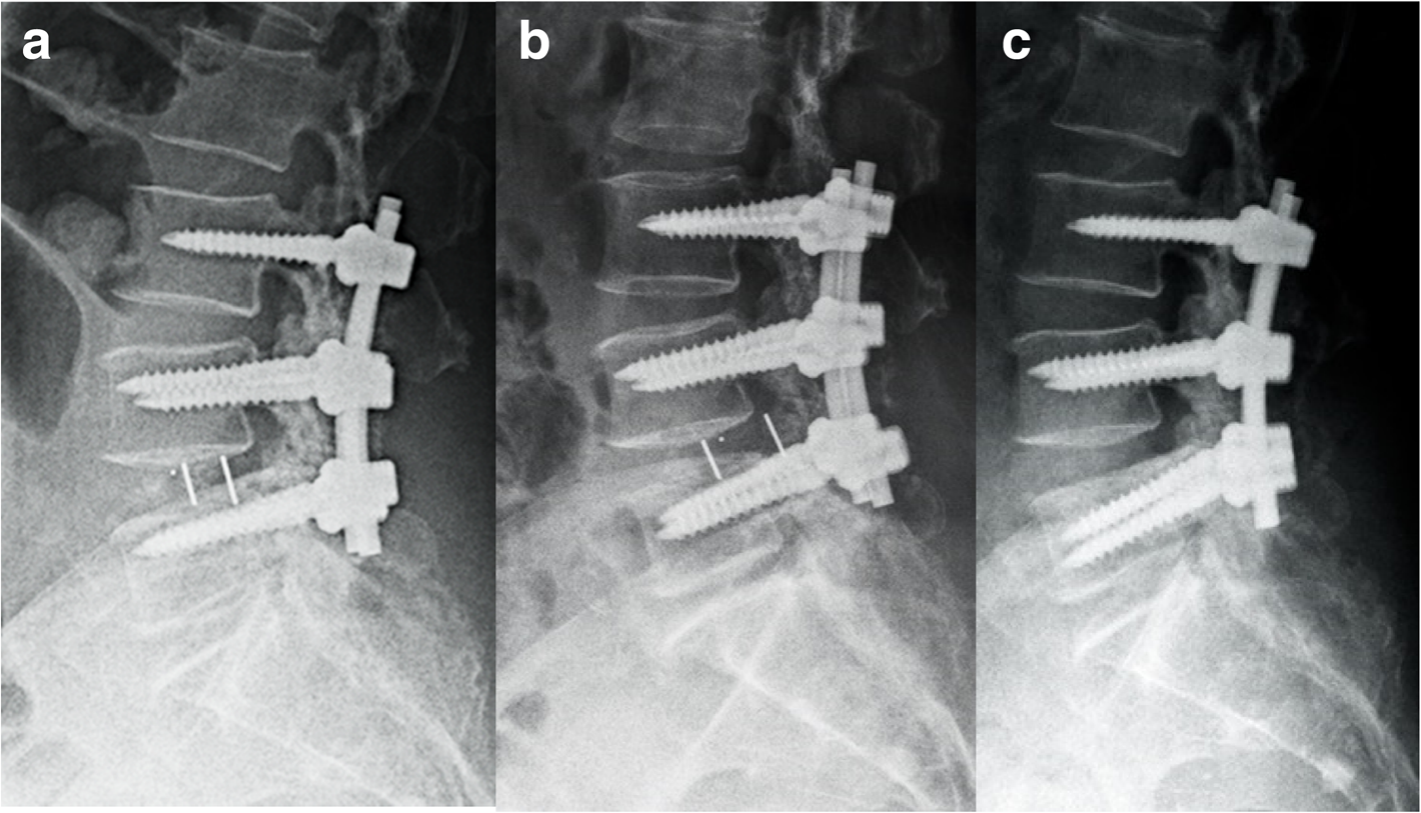
A 73-year-old female was diagnosed with degenerative spinal disease and was treated with L4/5 TLIF and L3-L5 bilateral posterior instrumentation. **a** Lateral radiograph 3 days postoperatively. **b** PCM at L4/5 13 days postoperatively. **c** Lateral radiograph obtained 2 days after revision surgery. The migrated cage was removed and the loosened right L5 pedicle screw was revised

There were 1151 patients in all who received open TLIF and bilateral pedicle screw instrumentation. Among the 198 (17.2%) excluded patients, 136 patients had postoperative follow-up of less than 24 months, 24 patients underwent a minimally invasive TLIF procedure, 15 patients developed deep SSI after operation, and 23 patients had dynamic fixation at one or more fusion levels. There were 953 patients (314 men and 639 women, mean age 62.65 ± 12.28 years) included in this study, and they were followed a mean of 38.06 ± 3.11 months after surgery. Twenty-four patients (2.52%, 8 male and 16 female, mean age 69.41 ± 6.96 years) developed PCM a mean of 4.92 ± 5.98 months after operation (Fig. 3). Six of these patients received revision surgery due to intolerable back pain, leg pain or progressive palsy (Fig. 4). The other 18 patients were asymptomatic or tolerable.

The mean fusion level was 2.19 ± 1.392. Cages were used in 1559 discs (L1/2: 20 discs; L2/3: 120 discs; L3/4: 356 discs; L4/5: 734 discs; L5/S: 329 discs). Compared to patients without PCM, we found longer fusion levels (*p* = 0.023) and older age (*p* = 0.006) in patients with PCM. Of the 21 patients with multilevel fusions (≥ 2 levels) in the PCM group, 15 patients (71.4%) had PCM occur at the end level of the fusion. The PCM incidences of kidney-shaped cages and bullet-shaped cages were 2.05 and 0.88% respectively, which was not statistically significant (*p =* 0.063)(Table 1). There was no statistically significant differences in diagnosis and cage insertion level between patients with and without PCM. The demographic data between randomly selected control group and patients without PCM showed no significant difference in terms of ages, gender, preoperative diagnosis or fusion levels.

**Table 1:** Characteristics of patients who developed PCM after TLIF among 953 Patients

	PCM	Non-PCM	Control	*P*-value
No.	24	929	100	
Age	69.41 ± 6.96	62.48 ± 12.34	61.92 ± 13.25	0.006
Sex(M/F)	8/16	306/623	35/65	1.000
Diagnosis				
SS	1	123	15	0.057
KS	7	96	21	
DS	13	590	45	
Revision	3	120	19	
Fusion levels	2.83 ± 1.37	2.17 ± 1.39	2.43 ± 1.55	0.023
Kidney/Bullet shaped cage	36/12 (18/6)^a^	842/669	90/73	0.063^b^
Level of cage inserted (level of PCM)				
L1/2	1	19	4	
L2/3	4 (1)	116	16	
L3/4	13 (3)	343	42	
L4/5	22 (17)	712	71	
L5/S1	8 (3)	321	30	

*PCM* posterior cage migration, *SS* spondylolytic spondylolisthesis, *KS* degenerative kyphosis or scoliosis, *DS* degenerative spondylolisthesis. The *p*-value is comparing PCM and non-PCM groups

^a^24 migrated cages from the PCM group

^b^Comparison of 24 migrated cages to other non-migrated cages

### Distribution of cage and PCM incidence by cage position

**Fig. 5:**
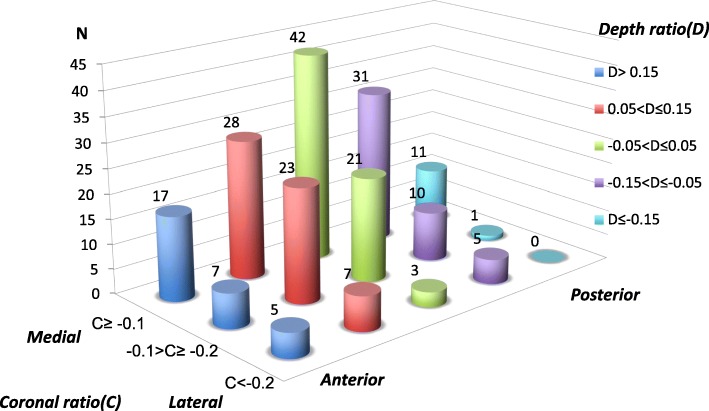
Distribution of all 211 cages in the PCM and control groups by cage position

**Fig. 6:**
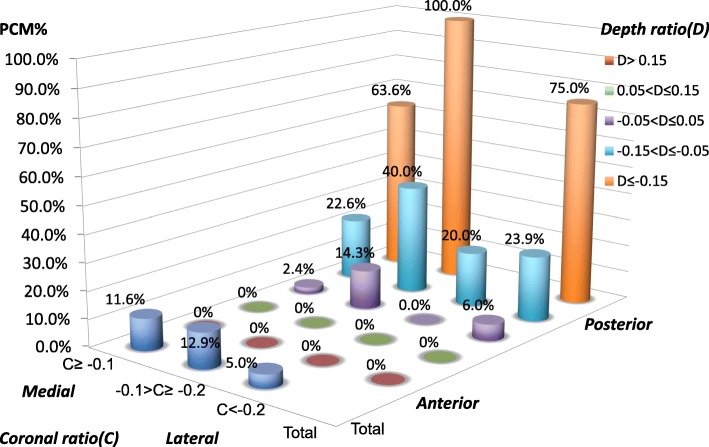
Distribution of PCM incidence in the PCM and control groups by cage position

There were 48 cages and 163 cages inserted in 24 patients in the PCM group and 100 patients in the control group respectively. Figure 5 displayed the distribution of all 211 inserted cages in the PCM and the control group by immediate postoperative cage position. Most cages (42 of 211, 19.90%) were located near the disc center with coronal ratio greater than − 0.1 and depth ratios lying between − 0.05 and 0.05. The distribution of PCM incidence by cage location demonstrated that no PCM occurred when the cage was located in the anterior disc space with a depth ratio larger than 0.05 (Fig. 6). The incidence of PCM increased from 0% (depth ratio > 0.15) to 75% (depth ratio ≤ − 0.15) as cages were placed more posteriorly. We didn’t find gradual increase or decrease in the incidence of PCM as cages were placed more medially in disc spaces. The incidence of PCM varied ranged from 5% (coronal ratio < − 0.2) to 12.9% (− 0.1 > Coronal ratio ≥ − 0.2).

### Migrated cages in the PCM group compared with the control group

Comparison of cage position, preoperative disc radiographic parameters, cage size and geometry between migrated cages in the PCM and control groups are listed in Table 2. Univariate analyses revealed significantly lower depth ratios (*p* < 0.001), larger preoperative DH (*p* < 0.001), greater preoperative disc sagittal translation (*p* < 0.05), and greater height variance (*p* < 0.001) in migrated cages. There was no significant difference in coronal ratio, preoperative disc ROM, slippage percentage, cage height, or cage shape between migrated cages in the PCM and control groups.

**Table 2:** Radiographic analysis between migrated cages in the PCM and control groups

	PCM group (*n* = 24)	Control group (*n* = 163)	*P*-value
Cage position			
Depth ratio	−0.119 ± 0.063	0.52 ± 0.107	< 0.001
Coronal ratio	−0.080 ± 0.062	− 0.103 ± 0.089	0.359
Preoperative disc radiographic parameter			
DH (mm)	11.43 ± 2.03	9.34 ± 2.54	< 0.001
ROM (°)	8.40 ± 5.38	6.38 ± 4.87	0.063
Translation (mm)	3.34 ± 2.45	2.11 ± 2.25	0.014
Slippage (%)	10.60 ± 10.54	7.32 ± 9.79	0.130
Cage size & geometry			
Cage height (mm)	11.17 ± 0.97	10.91 ± 1.15	0.302
Height variance^a^ (mm)	−0.26 ± 1.86	1.57 ± 2.36	< 0.001
Kidney/ Bullet shape	18/6	90/73	0.067

*DH* indicates disc height, *ROM* range of motion

^a^Height variance = cage height – preoperative disc height

### Migrated cages versus non-migrated cages in the PCM croup

Of the 48 cages inserted in 24 patients in the PCM group, 24 of 48 (50%) cages migrated posteriorly after the operation. Comparisons of cage position, preoperative disc radiographic parameters, cage size and geometry data between the migrated and non-migrated cages in the PCM group are listed in Table 3. The migrated cages were found to have significantly lower depth ratios (*p* < 0.001), larger preoperative disc heights (*p* < 0.001), larger cage heights (*p* < 0.05), and smaller height variance (*p* < 0.001). There were no significant differences in terms of preoperative disc ROM, slippage percentage, sagittal translation or cage shape.

**Table 3:** Radiographic analysis between migrated and non-migrated cages in the PCM group

	Migrated (*n* = 24)	Non-migrated (*n* = 24)	*P*-value
Cage position			
Depth ratio	−0.119 ± 0.063	0.022 ± 0.087	< 0.001
Coronal ratio	−0.080 ± 0.062	−0.086 ± 0.072	0.978
Preoperative disc radiographic parameter			
DH (mm)	11.43 ± 2.03	8.82 ± 2.53	< 0.001
ROM (°)	8.40 ± 5.38	6.73 ± 4.48	0.249
Translation (mm)	3.34 ± 2.45	3.19 ± 2.89	0.845
Slippage (%)	10.60 ± 10.54	11.23 ± 9.47	0.828
Cage size & geometry			
Cage height (mm)	11.17 ± 0.97	10.42 ± 1.018	0.009
Height variance^a^ (mm)	−0.26 ± 1.86	1.60 ± 2.65	< 0.001
Kidney/ Bullet shape	18/6	18/6	1.000

*DH* indicates disc height, *ROM* range of motion

^a^ Height variance = cage height – preoperative disc height

### Multivariate analysis

Fusion level, age, depth ratio, preoperative sagittal translation, and height variance were analyzed by the multivariate logistic regression model for all 211 cages in the PCM group and the control group. Height variance and preoperative DH were two related factors, and the former was chosen instead of the later in the model because height variance represented the effect of relative cage size to disc space. The model revealed that those with lower depth ratio (OR, 9.78E-4; 95% CI, 9.69E-4 - 9.87E-4; *p* < 0.001) and less height variance (OR, 0.757, 95% CI, 0.575–0997, *p* = 0.048) had a significantly higher risk of developing PCM.

## Discussion

Transforaminal lumbar interbody fusion via a fusion cage is widely performed to treat degenerative lumbar spinal disease. The cage acts as a spacer between vertebral endplates and provides mechanical support for the anterior column, leading to restoration of disc height and indirect decompression of the neuroforamen. Previous studies reported favorable clinical outcomes and fusion rates following TLIF via cages [14–16]. However, implantation of a single cage on one side can result in certain side effects. Despite the advantages, nerve root injury, dura mater injury, and PCM are possible complications that would result in spinal canal or exiting root compression, leading to low back pain, leg pain, or pseudoarthrosis [2, 7–9].

In the current study, we found that 24 out of 953 patients developed PCM in 4.92 ± 5.98 months (3 days - 22 months) after surgery. The prevalence of PCM was 2.52%, and one fourth of the patients (6 of 24) received revision surgery due to severe symptoms. The incidence was compatible to prior studies that reported the prevalence of PCM after TLIF or PLIF ranging from 1.17 to 14.7%, and occurred within 7 months after operation [3, 4, 6, 10]. Most cases of PCM occurred in the early postoperative period before achieving solid fusion. This complication could lead to devastating results and require revision operation for symptomatic patients.

Our hypothesis that cage position would affect PCM incidence was verified. The incidence of PCM increased as cages were placed more posteriorly among 211 cages in the PCM and control groups in the present study (Fig. 6). Posteriorly located cages were shown to be a significant risk factor for PCM in univariate and multivariate analyses. However, the cage coronal position showed no significant impact on PCM. To the best of our knowledge, this study is the first study that demonstrated a significant relationship between sagittal cage position and PCM.

A prior study from Aoki et al followed 125 patients with 4 PCM cases after receiving TLIF with radiopaque cages [3]. They measured the distance between the cage posterior margin and posterior margin of the cranial (or caudal) endplate. The migrated cages were found to have more posterior initial position than non-migrated cages. However, the difference was not significant. The authors failed to demonstrate the relationship between PCM and posteriorly located cages, possibly due to the small sample size. This current study had more patients compared to Aoki et al (24 patients versus 4 patients) and other existing literatures [3, 4, 6–8, 10]. Because the cages used in this study were all radiolucent, it was difficult to define cage margins by radiograph as Aoki et al did. Instead of measuring definite distance between the cage margin and the endplate margin, we plotted the cage center from the radiopaque markers, and calculated the depth ratio and the coronal ratio to demonstrate relative distance between the cage center and the disc center. As endplate length and width varies between individuals, we supposed the depth ratio and the coronal ratio were theoretically better methods for evaluating the influence of cage position on PCM.

With regard to biomechanical stabilization when comparing anteriorly located cages versus posteriorly located cages, a study of a synthetic spine model performed by Polly et al demonstrated constructs with anterior cage placement were significantly stiffer than constructs with central or posterior cage placement. The constructs with anterior cage placement had significantly reduced rod strain and increased cage strain in axial compression compared to constructs with posterior cage placement, which reinforced the concept of load sharing between anterior and posterior columns [5]. Following studies with cadaveric models by Faundez et al and Tallarico et al reported different results. The authors found no significant difference in flexion-extension ROM between anterior cage placement and posterior cage placement constructs [17, 18]. An in vivo kinematic study demonstrated a non-significant correlation (*p* = 0.055) between more anteriorly located cages and greater standing flexion-extension stability [19]. The results regarding construct stability were inconclusive. The remnant thick anterior annulus fibrous after TLIF could support the anterior column and influence overall stability, which might make the impact of sagittal cage positioning less distinguishable. However, in regard to pressure between the endplate and cage, the cage shares more load when placed more anteriorly in the disc space. Compared to posteriorly located cages, we supposed that anteriorly located cages bear more pressure as gravity transmits and generates greater friction to resist posterior migration.

To investigate the influence of coronal cage position on construct stability, Quigley et al utilized a synthetic spine fusion model and compared constructs with anterolateral, central, or anterior cage placement [20]. The authors found no significant difference in strain, load-to-failure, or cage displacement after long-term cycling. Comer et al also compared a cadaveric model with centrally placed and laterally placed cage constructs [21]. Despite significantly lower stiffness under compression, constructs with lateral cage placement demonstrated similar stiffness to centrally positioned ones in flexion, extension, lateral bending, and torsion. Thus, it is theorized that the coronal position of the cage has less impact on PCM.

To minimize the risk of PCM, we should avoid placing the cage with its center located posterior to the disc center. However, due to limited discectomy by TLIF approach [22], the remnant disc and the bone graft packed in the disc space may hinder surgeons from placing the cage center anteriorly enough to reach or pass the disc center. The technique of disc preparation plays an important role in avoiding PCM. Before cage insertion, the trial implant should reach adequate depth after disc preparation and bone graft packing. This process would allow surgeons to insert the cage along the path created by trial implant and achieved an ideal cage position. It is always necessary to monitor cage position during surgery by fluorescent imaging. The depth ratio in lateral radiographs is a useful tool to detect mal-positioned cages before wound closure.

In the current study, PCM was found in discs with significantly increased preoperative disc height. Height variance, which was defined as ‘cage height - preoperative disc height’, represented the relative size of the cage in the disc space. Less height variance was found to be a significant risk factor in the univariate and multivariate analyses, which verified the hypothesis that undersized cages are a risk factor for PCM. Multiple studies reported similar results. Kimura et al analyzed 1070 cases (9 with PCM) retrospectively and found significantly larger preoperative disc height in migrated cages [6]. Li et al and Aoki et al reported undersized cages to be a significant risk factor for PCM [3, 10]. Larger cage height creates more tension in peri-vertebral soft tissue, resulting in higher contact pressure at endplate-cage interfaces. The interfaces are able to provide more friction during motion to resist posterior migration. We recommend using cages with heights equal to or larger than preoperative disc heights.

Cage geometry was not verified to influence PCM in the present study. However, kidney-shaped cages had higher PCM incidence than bullet-shaped cages, even though this difference was not statistically significant (*p* = 0.063). Seventy-five percent of migrated cages (18 of 24) were kidney-shaped, while only 56.32% of inserted cages were kidney-shaped in the current study. We hypothesized that the non-significant difference resulted from the cage insertion technique. Kidney-shaped cages are designed to rotate approximately 90 degrees within the disc space during insertion. Therefore, the cage moves in a curved trajectory during insertion, making it relatively difficult to drive the whole cage anteriorly when the bone graft is packed in the anterior disc space. For bullet-shaped cage, the cage moves anteriorly in a straight trajectory during insertion and takes less effort to reach central or anterior disc space. Furthermore, less effort is required to withdraw the bullet-shaped trial implant from within the disc space, which may make surgeons choose larger implants to achieve enough tension. It is theorized that these differences during insertion make it less possible to have undersized bullet-shaped cages located in posterior disc space.

Prior biomechanical studies showed no difference in stability between kidney-shaped and bullet-shaped cage constructs [21]. Reports from Aoki et al mentioned that all of their migrated cages were bullet-shaped cages [3]. Pan et al and Zhao et al reported bullet-shaped cages had significantly higher PCM incidence [4, 7]. However, there was no comparison to kidney-shaped cages from Aoki et al [3], and all these studies were analyses of less than 10 patients with PCM. Our results suggest cage geometry has less influence on PCM, and both kidney-shaped and bullet-shaped cage designs were viable option when surgeons inserted an implant with appropriate size and enough depth.

Segmental instability of the lumbar spine resulting from degenerative or spondylolytic spondylolisthesis are common indications for spinal fusion surgery. We examined the influence of segmental stability on PCM and measured several radiographic parameters in this series. There was significantly more sagittal translation in discs developing PCM compared to the control group in the univariate model. However, there was no significant difference between discs with migrated and non-migrated cages in the PCM group. The multivariate logistic model showed non-significant results for sagittal translation. Previous studies also reported conflicting results. Aoki et al found no significant difference in translation, slippage or ROM, while Kimura et al reported more ROM in discs with PCM [3, 6]. We speculated that it was not slippage percentage in standing lateral radiographs, but sagittal translation in dynamic images that might influence PCM. Further studies with larger sample sizes are needed to verify this hypothesis.

Significantly longer fusion levels were found for patients with PCM in univariate analysis, while fusion level was a non-significant factor in the multivariate logistic model. In the present study, we found that, for patients receiving multilevel fusion in the PCM group, PCM occurred mostly (15 of 21, 71.43%) at the end level of the fusion. As gravity transmits vertically, there is large cantilever bending torque applied at the end levels, possibly resulting in a higher failure rate. Kimura et al reported significantly longer fusion levels in all 9 cases with PCM. All of them were multilevel fusions, and PCM occurred at the end disc [6]. Based on these results, more caution is required for multilevel fusions, and additional effort should be put into end level stability.

There are several limitations in this study. First, although this study had more patients with PCM than any other study, the number of patients was still relatively small. Second, the study was conducted retrospectively without randomization. Third, large proportions of patients (11.82%) were excluded due to short follow-up, even though all of their latest radiographs showed stable cages without signs of posterior migration. Fourth, in addition to cage position and cage height, the spine sagittal alignment parameters are possible risk factors that yet to be determined. These parameters were not included in this study because whole spine radiographs were not available during the period of case collection. Further study is needed to investigate the role of sagittal alignment parameters in development of PCM.

## Conclusion

From this retrospective study, we found that posteriorly located cages and undersized cages were two major risk factors for posterior cage migration. Longer fusion levels, preoperative spine mobility, coronal cage position, and cage shape design (kidney or bullet-shaped) were found to be non-significant risk factors for PCM. These complications could lead to poor outcomes and cause revision surgery. For spine surgeons, it is important to avoid placing the cage center posterior to the disc center, and cage height should be equal to or larger than preoperative disc height.

## Abbreviations

AP: Anteroposterior
DH: Disc height
MRI: Magnetic resonance imaging
PCM: Posterior cage migration
PEEK: Polyetheretherketone
PLIF: Posterolateral lumbar interbody fusion
ROM: Range of motion
SSI: Surgical site infection
TLIF: Transforaminal lumbar interbody fusion
